# Macroalgae of the Campeche Bank, Gulf of Mexico

**DOI:** 10.3897/BDJ.12.e141321

**Published:** 2024-12-17

**Authors:** Ileana Ortegón-Aznar, Ana M. Suárez, Beatriz Martínez-Daranas, Mariana Álvarez-Rocha, Carmen Galindo-De Santiago, Raúl E. Castillo-Cupul, Nuno Simões

**Affiliations:** 1 Universidad Autónoma de Yucatán, Mérida, Mexico Universidad Autónoma de Yucatán Mérida Mexico; 2 Centro de Investigaciones Marinas, Universidad de La Habana, La Habana, Cuba Centro de Investigaciones Marinas, Universidad de La Habana La Habana Cuba; 3 Instituto de Ciencias del Mar y Limnología, Puerto Morelos, Mexico Instituto de Ciencias del Mar y Limnología Puerto Morelos Mexico; 4 Escuela Nacional de Estudios Superiores, Unidad Mérida, UNAM, Ucú, Yucatán, Mexico Escuela Nacional de Estudios Superiores, Unidad Mérida, UNAM Ucú, Yucatán Mexico; 5 Unidad Multidisciplinaria de Docencia e Investigación Sisal, Facultad de Ciencias, UNAM, Yucatán, Mexico Unidad Multidisciplinaria de Docencia e Investigación Sisal, Facultad de Ciencias, UNAM Yucatán Mexico; 6 KALANBIO A.C. Research for Conservation, Mérida, Mexico KALANBIO A.C. Research for Conservation Mérida Mexico; 7 Universidad Nacional Autonoma de Mexico, Mexico, Mexico Universidad Nacional Autonoma de Mexico Mexico Mexico; 8 Laboratorio Nacional de Resiliencia Costera, Mexico, Mexico Laboratorio Nacional de Resiliencia Costera Mexico Mexico; 9 International Chair for Coastal and Marine Studies, Harte Research Institute for Gulf of Mexico Studies, Corpus Christi, United States of America International Chair for Coastal and Marine Studies, Harte Research Institute for Gulf of Mexico Studies Corpus Christi United States of America

**Keywords:** biodiversity, seaweeds, reefs

## Abstract

**Background:**

The coastal habitats in the southern Gulf of Mexico face multiple threats, such as rising water temperatures, acidification, increased turbidity, invasive species and pollutants. This imperils the biodiversity of beaches, wetlands and coral reefs. To address this, there is a need for comprehensive baseline information on marine biodiversity. Several reefs in the Gulf of Mexico have been extensively studied, yet smaller reefs on the Yucatan continental shelf lack thorough exploration despite their ecological significance. These reefs serve as crucial biodiversity hotspots influenced by environmental characteristics, receiving diverse taxa from the Gulf of Mexico.

The macroalgae study at the Bank of Campeche dates back to the 1950s, but comprehensive investigations have been sporadic. The recent study aims to fill this gap, contributing to the taxonomic inventory of the area's benthic macroflora. Methodologically, extensive sampling across eight reefs was conducted, collecting and preserving macroalgae samples for identification in the laboratory.

The study documented 90 infrageneric taxa across the reefs, with Cayo Arenas exhibiting the highest species count. Additionally, three new distribution reports for Mexico were identified in the region. The distribution of species varied amongst locations, with few species in common even amongst geographically proximate reefs. The diversity found in these reefs slightly trails behind other studied regions, but surpasses previous reports for the Campeche Bank.

It is important to emphasise that the significance of this study lies in its focus on remote reefs with complicated and costly access logistics. Additionally, it is one of the first publicly available datasets published for this region.

The study aligns with existing literature on prevalent families in reef environments and highlights differences in species distribution, based on depth variations amongst reefs. The findings of new distribution records in the region and the distinctiveness of localities despite their proximity underscore the unique ecological dynamics of these reefs.

**New information:**

These reefs are located in remote and difficult-to-access areas, highlighting the importance of the data obtained on their biodiversity and conservation status. This work presents for the first time a list of macroalgae for the reefs of Banco Nuevo, Banco Obispo, Banco Pera and Serpientes Reef. Three new distribution reports for Yucatan were identified at the Banco Obispo reef: *Botryocladiashanksii* E. Y. Dawson, *Ceratodictyonscoparium* (Montagne & Millardet) R. E. Norris and *Asteromeniapeltata* (W. R. Taylor) Huisman & Millar and a new report for the Mexican Atlantic, *Herposiphoniaparca* Setchell at the Triángulo Oeste reef. This results in distinct algal communities compared to other reefs in the region, emphasising their ecological significance and the need for continued research and conservation efforts. To our knowledge, this is one of the first interoperable datasets being published on the marine algae of the southern Gulf of Mexico reef ecosystems.

## Introduction

The habitats of coastal areas are subjected to a variety of anthropogenic and environmental pressures, which endanger the biological diversity that inhabits them. Amongst the most exposed ecosystems are beaches, coastal wetlands and coral reefs, for which the expected increase in water temperature, acidification due to the effects of global climate change, increased turbidity due to sediments and the presence of invasive species could have negative and high-impact effects in the medium and long term (Cooley et al. 2022). This scenario makes it imperative to compile reference information to establish the current conditions of marine biodiversity and to understand the current state of these ecosystems in the southern Gulf of Mexico. Knowledge of the diversity, abundance and distribution of species is the foundation for the development of conservation and natural resource utilisation projects aimed at preserving their ecological, economic and cultural importance.

The Gulf of Mexico platform has many important reef systems that have been extensively studied (Chávez-Hidalgo A et al. 2008, Horta-Puga et al. 2015). However, there is a lack of information about many smaller reefs located on the continental shelf of Yucatan, despite being important centres of biodiversity and fisheries resources (Zarco-Perelló et al. 2013).

The reefs of the Campeche Bank in the Gulf of Mexico, such as Alacranes Reef, Cayo Arenas, Cayo Arcas and Triangulos, have been the subject of various studies (Jordán-Dahlgren 2002, Tunnell et al. 2007, Zarco-Perelló et al. 2013). Nevertheless, the study of macroalgae in these environments has been scarce and discontinuous.

The first studies of macroalgae on the Campeche Bank were conducted in the 1950s, Huerta-Múzquiz collecting samples at Cayo Arenas and Alacranes Reef and identifying 19 and 21 infrageneric taxa, respectively (Huerta 1958). Subsequently, in 1960, in the eulittoral zone around Isla Pérez, 77 infrageneric taxa were identified (Huerta 1961). There are also records of collections by H. J. Humm in the Campeche Bank (Humm 1952) and reports of 17 algal species in the work of Kornicker et al. (1959). Some of the more extensive studies are those of Kim (1964), who characterised the phycoflora of Alacranes Reef with 198 infrageneric taxa and Huerta-Múzquiz et al. (1987), who compiled advances in the study of marine algae of the Yucatan Peninsula, listing 412 infrageneric taxa (140 Chlorophyta, 56 Phaeophyceae, 199 Rhodophyta and 17 Cyanobacteria) and reported algal records in Alacranes Reef, Cayo Arenas and Triangulo Oeste. For the Madascar reefs at Sisal, Yucatan, there is only the work of Ortegon-Aznar et al. (2008), who report 55 taxa of macroalgae. The most recent study reported 22 species for Alacranes, six species for Cayo Arcas and 16 species for Bajo Obispo (González-Solis et al. 2018).

Therefore, this study aims to contribute to the knowledge of macroalgae in the reef system of the Campeche Bank in the Gulf of Mexico by providing information on the composition and richness of benthic macroalgae communities.

## Project description

### Title

Benthic macroflora of the reef system of the Campeche Bank

### Personnel

This study is part of a megaproject with the objective of generating a taxonomic inventory of the fauna and benthic macroflora of the reef system of the Campeche Bank. It aims to create a georeferenced dataset of marine biota presence/absence that follows the DarwinCore data standard (DwC) uploaded at the Caribbean OBIS Node. Additionally, it seeks to contribute to strengthening the information on biodiversity species for the Gulf of Mexico at the National Biodiversity Information System (SNIB) of CONABIO, as well as national and regional collections, updating, completing and identifying information gaps in floristic inventories and taxonomic authority catalogues.

## Sampling methods

### Study extent

**Figure 1.**
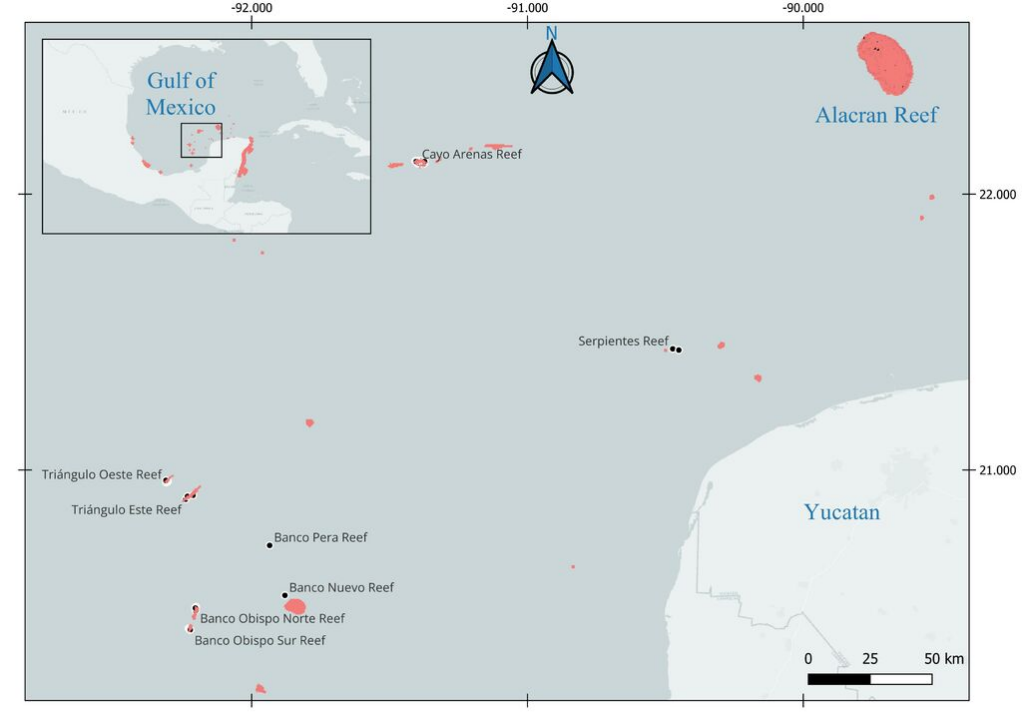
Map of the studied Reef Systems in the Gulf of Mexico. The black dots represent the sampled locations and the red colour shows the distribution of existing reefs in the Gulf of Mexico.

Sampling was conducted during three excursions in 2017 to eight reefs of the Campeche Bank: Arrecife Banco Nuevo (BN), Arrecife Banco Obispo Norte (BON), Arrecife Banco Obispo Este (BOE), Arrecife Banco Pera (BP), Cayo Arenas Reef West Triangle Reef (TO), East Triangle Reef (TE) and Serpientes Reef (Ser) (Fig. 1). The first excursion took place at Cayo Arenas (20 May to 28 May 2017), the second at the submerged Serpientes Reef (29 July 2017) and the last one at the submerged cays in the West (8-16 September 2017). At each site, the geographic coordinates of each sampling site were physically located using a Garmin© GPS (Global Positioning System) with an accuracy of ±4 m (Table 2). Sampling was conducted using self-contained underwater breathing apparatus (SCUBA) diving, free diving and general intertidal collection methods. Macroalgae were collected and, in the case of specimens adhering to rocks, a spatula or knife was used. The organisms were placed in sealed bags with seawater at each sampling site and station. Each sample is associated with collection information recorded in the field in the form of a label containing the following information: identification of the material at the highest possible taxonomic level in the field, location (with geographic coordinates), depth, type of substrate or environment, date and time of collection, collector's name, collection code, photograph code and collection method used.

### Sampling description

For Cayo Arenas, 31 samplings were conducted, including 30 daytime samplings and one night-time dive. Organism collections were made on the coral reefs around the sandy cay and adjacent reef areas in benthic ecosystems considered as shallow waters (i.e. depths between 0 and 20 m). A total of 55% of organism collections were made using SCUBA, 29% were conducted in the intertidal zone through beach samplings and 16% were collected through free diving. During the campaign, 92 algal samples were collected from various types of environments and environmental data were recorded for each sampling point, including salinity and temperature data.

For the submerged cays sampling, a total of 79 samplings were conducted by four different working groups across six different reef areas, comprising 74 daytime samplings and five night-time dives. Organism collections were carried out on the coral reefs, specifically on TW and TE reefs, around sandy cays, in shallow benthic ecosystems considered shallow waters (i.e. depths between 0 and 30 m) and on the submerged ship found at point 11_TW. Approximately 208 algal specimens were collected during 79 dives, one snorkelling excursion and one intertidal collection, covering 61 different sites within six reefs located to the west of the Campeche Bank. Only one sampling was conducted in the intertidal zone using free diving (at Triángulos Este Reef), while the rest of the collections were made using SCUBA.

For the Serpientes Reef sampling, two samplings were conducted, one dive at each site on the reef peaks at depths of 8 to 13 m. A total of 29 batches with 41 algal organisms were collected.

In the laboratory, the samples were separated and preserved in formalin (4%). In some cases, pressed vouchers were created and deposited in the Algae section of the Herbarium of the Faculty of Sciences of UNAM (FCME). The pressed vouchers were consecutively assigned numbers: GM-925 to GM-1204 and they can be consulted in the dataset previously uploaded to the Ocean Biodiversity Information System (OBIS). Subsequently, identification was performed at the best possible taxonomic level by observing complete thalli and making microscopic slides using a stereoscope and an optical microscope. The main literature used for identification included the identification guides by Littler and Littler (2000) and Dawes and Mathieson (2008). Taxonomic nomenclature was verified using the Algaebase database (Guiry and Guiry 2024).

## Geographic coverage

### Description

Western submerged cays (Banco Obispo Triángulos Oeste and Este, Banco Pera and Banco Nuevo) Arrecife Serpientes, and Cayo Arenas at the Campeche Bank.

### Coordinates

−90.45086 and −92.31128 Latitude; 22.12092 and 20.42059 Longitude.

## Taxonomic coverage

### Description

In this study, 90 infrageneric taxa were identified at the species level, which are located in two kingdoms: Plantae and Cromista and belong to the three phyla: Chlorophyta, Rhodophyta and Heterokontophyta, which contain five classes, 16 orders, 37 families and 62 genera of macroalgae (Table 1).

### Taxa included

**Table:** 

Rank	Scientific Name	Common Name
kingdom	Plantae	Plant
phylum	Chlorophyta	Green Algae
class	Ulvophyceae	
order	Cladophorales	
order	Bryopsidales	
order	Siphonocladales	
family	Anadyomenaceae	
family	Bryopsidaceae	
family	Caulerpaceae	
family	Boodleaceae	
family	Siphonocladaceae	
family	Halimedaceae	
family	Udoteaceae	
family	Valoniaceae	
phylum	Rhodophyta	Red Algae
class	Florideophyceae	
class	Stylonematophyceae	
class	Compsopogonophyceae	
order	Ceramiales	
order	Corallinales	
order	Bonnemaisoniales	
order	Rhodymeniales	
order	Nemaliales	
order	Erythropeltales	
order	Gigartinales	
order	Stylonematales	
family	Ceramiaceae	
family	Lomentariaceae	
family	Galaxauraceae	
family	Dasyaceae	
family	Erythrotrichiaceae	
family	Solieriaceae	
family	Gelidiellaceae	
family	Gelidiaceae	
family	Wrangeliaceae	
family	Rhodomelaceae	
family	Corallinaceae	
family	Cystocloniaceae	
family	Delesseriaceae	
family	Liagoraceae	
family	Pterocladiaceae	
family	Stylonemataceae	
kingdom	Chromista	
phylum	Heterokontophyta	
class	Phaeophyceae	Brown Algae
order	Dictyotales	
order	Ectocarpales	
order	Fucales	
family	Dictyotaceae	
family	Acinetosporaceae	
family	Sargassaceae	

## Temporal coverage

**Formation period:** 20-5-2017; 16-9-2017.

### Notes

Data were collected from 20-5-2017 to 16-9-2017, at Cayo Arenas from 20 - 28 May 2017. Data were collected at Serpientes Reef on 29 July 2017. Data were collected at Triángulo Oeste, Triángulo Este, Banco Obispo Norte, Banco Obispo Sur, Banco Nuevo and Banco Pera from 8 - 16 September 2017.

## Collection data

### Collection name

Within theHerbarium of the Faculty of Sciences of UNAM (FCME), the pressed vouchers were consecutively assigned numbers: GM-925 to GM-1204 and they can be consulted in the dataset previously uploaded to the Ocean Biodiversity Information System (OBIS).

### Collection identifier

the pressed vouchers were consecutively assigned numbers: GM-925 to GM-1204.

### Parent collection identifier

can be consulted in the dataset previously uploaded to the Ocean Biodiversity Information System (OBIS).

### Specimen preservation method

collection is in formalin 4%.

### Curatorial unit

GM-925 to GM-1204,

## Usage licence

### Usage licence

Other

### IP rights notes

CC BY-NC 4.0

## Data resources

### Data package title

Macroalgae from eight reefs in the Campeche Bank, collected by the National Autonomous University of Mexico (UNAM)

### Resource link

https://doi.org/10.15468/k4u4mx

### Alternative identifiers

https://obis.org/dataset/1de8b1fe-8847-4e4f-9140-cfa6dab91e4c

### Number of data sets

1

### Data set 1.

#### Data set name

Macroalgae from eight reefs in the Campeche Bank, collected by the National Autonomous University of Mexico (UNAM).

#### Data format

CSV

#### Download URL

https://ipt.iobis.org/caribbeanobis/archive.do?r=macroalgae_of_the_campeche_bank

#### Description

The dataset represents the macroalgal composition of eight reefs in the Campeche Bank: Banco Nuevo Reef (BN), Banco Obispo Norte Reef (BON), Banco Obispo Sur Reef (BOS), Banco Pera Reef (BP), Cayo Arenas Reef, West Triangle Reef (TO), East Triangle Reef (TE) and Serpientes Reef (Ser). The dataset contains a total of 395 records, which are taxonomically classified as follows: two at the phylum level, four at the subclass level, four at the order level, three at the family level, 185 at the genus level and 197 records at the species level. In this work, as we only put the different infrageneric taxa identified, there is a differentiation with the dataset because it contains the total number of specimens that were identified at the species level, per specimen; therefore, 195 taxa identified at the species level are reported and 185 were identified at the genus level.

**Data set 1.** 

Column label	Column description
occurrenceID	Biological record ID.
basisOfRecord	Origin or specific evidence from which the organism/sample is derived.
type	Type of evidence that gives rise to the record.
institutionCode	The full name of the institution holding the specimen or record information.
collectionCode	The name, acronym, alphanumeric code or initials that identify the collection or dataset from which the organism comes.
catalogNumber	An identifier assigned to the specimen, sample or lot in the biological collection.
datasetName	The name of the dataset from which the biological record is derived.
language	The language of the dataset.
recordedBy	The collector or main observer.
recordedByID	ID of the people (observers or collectors), groups or organisations responsible for carrying out the registration (ORCID).
occurrenceStatus	State that accounts for the presence or absence of a taxon at a location.
preparations	Preparations and methods of conservation of a specimen or a sample of the specimen.
disposition	The current status of a specimen in relation to the collection identified in collectionCode or collectionID.
samplingProtocol	The name, description or reference of the sampling method or protocol used to perform the sampling.
eventDate	Sampling or observation date.
year	Year of sampling and observation.
month	Month of sampling or observation.
day	Sampling or observation day.
habitat	Description of the habitat in which the event occurred.
fieldNumber	An indicator about the existence of or reference to field notes.
continent	The name of the continent on which the location takes place.
waterBody	The name and type of the body of water in which the location takes place.
country	The country name of the location.
countryCode	The standard code for the country of the location.
locality	The most location-specific geographic information.
maximumDepthInMetres	The greatest depth of a depth range below the local surface.
minimumDepthInMetres	The lowest depth of a depth range below the local surface.
decimalLatitude	The geographic latitude (in decimal degrees, using the spatial reference system provided in geodeticDatum).
decimalLongitude	The geographic longitude (in decimal degrees, using the spatial reference system provided in geodeticDatum).
geodeticDatum	The ellipsoid, geodetic datum or spatial reference system (SRS) on which geographic coordinates are based.
coordinateUncertaintyInMetres	The horizontal distance (in metres) of the decimalLatitude and decimalLongitude provided describing the smallest circle containing the entire location.
identifiedBy	Names of the people responsible for identifying the organism.
identifiedByID	ID of the persons responsible for identifying the organism (ORCID).
dateIdentified	The date on which the observation, collection or sample was taxonomically identified.
identificationRemarks	Comments or notes on identification.
identificationQualifier	The degree of uncertainty of the identification can be indicated by adding various terms, such as aff. and cf. to the scientific name.
scientificName	The canonical scientific name with the authorship corresponding to the taxonomic category to which the determination of the observed or collected organism was achieved.
scientificNameID	An identifier of nomenclature details (non-taxonomic) according to the scientific name documented in the scientificName element.
kingdom	The name of the kingdom to which the taxon belongs.
phylum	The name of the phylum or division to which the taxon belongs.
class	The name of the class or division to which the taxon belongs.
order	The name of the order to which the taxon belongs.
family	The name of the family to which the taxon belongs,
genus	The name of the genus to which the taxon belongs.
taxonRank	the taxonomic category of the most specific name present in the scientificName.

## Additional information

To complement and make this study clearer, two subsections are attached as additional information, one for results and another for discussion and conclusions.

### Results

A total of 395 algae samples were gathered across eight reefs of the Campeche Bank around the sandy cays. Arrecife Banco Nuevo (BN) had 10 species from 17 samples, Arrecife Banco Obispo Norte (BON) nine species from 24 samples, Arrecife Banco Obispo Este (BOE) 20 species and Arrecife Banco Pera (BP) 24 species. Furthermore, Cayo Arenas Reef contributed a total of 28 species from 94 samples, West Triangle Reef (TO) 23 species, East Triangle Reef (TE) 15 species and Serpientes Reef (Ser) 18 species. In total, 90 species, 66 genera, 36 families, 16 orders, five classes and three phyla were identified (Table 1). Amongst these, 32 species belonged to Chlorophyta, 39 to Rhodophyta and 19 to Phaeophyceae. Cayo Arenas had the highest species count, featuring 28 species – 11 Chlorophyta, eight Rhodophyta and nine Phaeophyceae. In contrast, Banco Obispo Norte Reef had the lowest species with nine species: four Chlorophyta, four Rhodophyta and one Phaeophyceae (Fig. 3).

**Figure 2.**
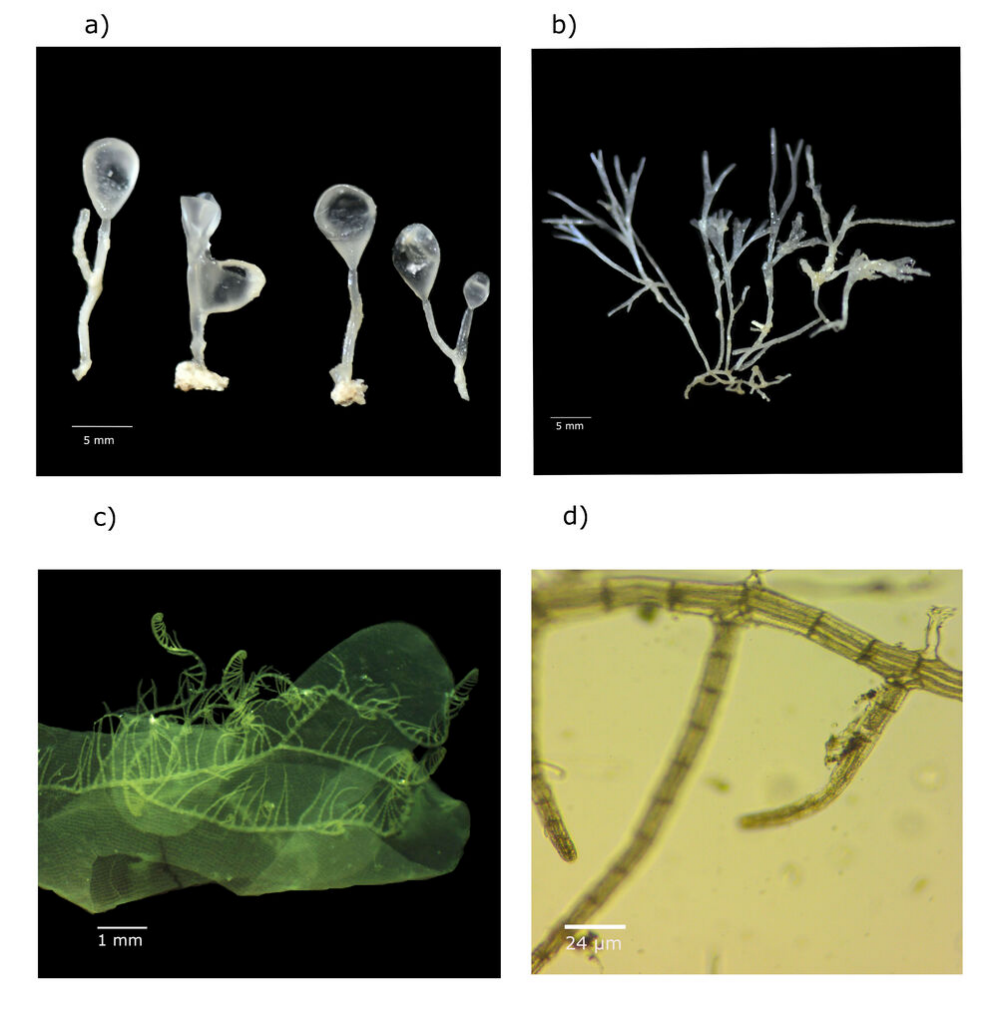
New records: **a** *Botryocladiashanksii* E. Y. Dawson, 1962 (GM-1082) Banco Obispo Sur Reef; **b** *Ceratodictyonscoparium* (Montagne & Millardet) R.E.Norris, 1987 (GM-1004) Triángulo Oeste Reef; **c-d** *Herposiphoniaparca* Setchell, 1926 (GM-1186) Triángulo Oeste Reef.

**Figure 3.**
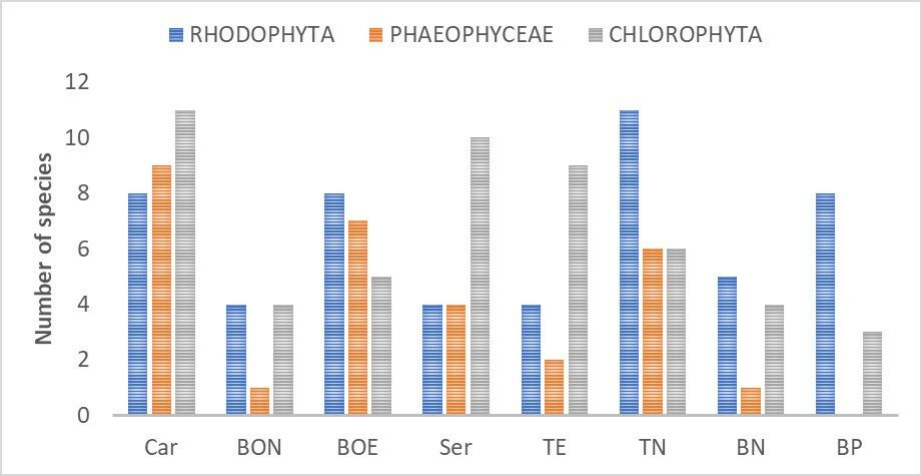
Species richness by phylum and locations (Car = cayo Arenas, BON = Banco Obispo norte, BOE = Banco Obispo Este, Ser = Serpientes, TE = Triangulos Este, TN = Triangulos Norte, BN: Bajo Nuevo, BP: Banco Pera).

Four species were newly reported in this region: *Asteromeniapeltata* (W. R. Taylor) Huisman & A. J. K. Millar, *Botryocladiashanksii* E. Y. Dawson, 1962, *Ceratodictyonscoparium* (Montagne & Millardet) R. E. Norris, 1987 and *Herposiphoniaparca* Setchell, 1926 (Fig. 2).

*Asteromeniapeltata*: This algae species has a thalli adhering to the substrate by small attachment discs. Plants with stipitate stems and peltate or lobed dorsiventral laminae.The laminae widen to reach 4 cm in width and can be simple or irregularly lobed circles; often the laminae anastomose with each other at the margins. In their natural environment, plants are iridescent with colours ranging from yellow to reddish-brown (Mendoza-González and Mateo-Cid 2007). Its distribution spans Mexican Caribbean, Caribbean Islands such as Bahamas and Cuba and parts of the Western Atlantic and South America including Colombia and Venezuela (Guiry and Guiry 2024).

*Botryocladiashanksii*: Is characterised by its red hue, grows epilithically, forming large clumps up to 26 mm tall. These plants are attached by a discoid holdfast, producing one or two solid terete axes (around 0.5 mm in diameter). These axes are dichotomously branched 1–2 times, bearing up to ten lateral vesicles confined to their distal parts (Afonso Carrillo and Sobrino 2004). Their distribution spans various regions, including Atlantic Islands like the Canary Islands, Central American areas like Belize and Costa Rica, Caribbean Islands like Cuba and parts of the Western Atlantic and South America, including Colombia (Guiry and Guiry 2024) (Fig. 2a).

*Ceratodictyonscoparium*: This species exhibits a tufted, dark red thallus that is notably tough, hard and wiry. It features creeping and erect axes, with the lower sections primarily terete. However, the erect axes, particularly distally, are distinctly flattened and irregularly subdichotomously branched or palmate. The axes possess a cartilaginous nature. Tetrasporangia are found within inflated nemathecia covering branch apices, typically divided cruciately, decussately or frequently in a tetrahedral manner. It grows epilithically in the subtidal zones. Its distribution spans Central America, including Panama; Caribbean Islands such as Cuba; and South America, notably Brazil (Huisman 2018) (Fig. 2B).

*Herposiphoniaparca*: This algae species has prostrated primary axes that produce erect, epiphytic, ecocutate branches, measuring 1–2 mm in height. These branches adhere to the substrate via unicellular rhizoids terminating in haptera, lacking an open connection to pericentral cells. The erect branches are sparsely branched, typically with 8–12 segments, each consisting of six to eight pericentral cells. The diameter of prostrate shafts ranges from 76–78 μm, while the diameter of erect shafts in the middle portion measures 58–67 μm. Apices feature vegetative trichoblasts. Tetrahedral tetrasporangia are formed in the distal portions of the determinate erect branches, arranged either in straight or spiral series. Terminal cistocars and spiral spermatotial branches are formed on the apices of the determinate branches (Garcia et al. 2008). Its distribution includes North America, particularly Florida; Central America, encompassing Belize; Caribbean Islands like Cuba; and regions across the Western Atlantic and South America, including Brazil, Colombia and Venezuela (Guiry and Guiry 2024) (Fig. 2C and D).

Each location exhibited its own set of specific species and, while some species were shared amongst them, the most prevalent species (comprising 55% of the findings) across the localities was *Caulerpaverticillata*. This was followed by *Caulerpamicrophysa* (*Weber van Bosse*) *Feldmann*, *Dictyotapulchella* Hörnig & Schnetter, *Stypopodiumzonale* (J.V. Lamouroux) Papenfuss and *Ceratodictyonscoparium*, which collectively constituted 44% of the species in all the localities. Additionally, species like *Amphiroatribulus* (J. Ellis & Solander) J. V. Lamouroux, *Hypneaspinella* (C. Agardh) Kützing, *Dictyotabartayresiana* J. V. Lamouroux, and *Anadyomenesaldanhae* Joly & Oliveira, accounted for 33% of the observed species.

### Discussion and conclusion

The diversity found within these reef banks, totalling 90 infrageneric taxa, slightly trails behind the findings in Sisal reefs, which recorded 123 species (Vilchis et al. 2024). Nevertheless, this count surpasses the previous report on five Campeche Bank reefs, where the highest species count was 21 at Bajo Obispo (González-Solis et al. 2018). This discrepancy is likely attributed to variations in sampling techniques and sampling effort. Interestingly, *C.verticillata*, the species with the highest relative frequency across five out of eight localities (Table 2), was not documented in the last floristic study of the Bank of Campeche (González-Solis et al. 2018). However, previous studies have indicated its abundance in Sisal reefs, along with *H.spinella*, both of which are macroalgae commonly found in reef ecosystems (Littler and Littler 2000).

At the family level, the findings align with existing literature (Dreckmann 1998, Mendoza-González et al. 2016, Vilchis et al. 2018, García García et al. 2020, Pedroche and Sentíes 2020, Ortegón-Aznar and León-Tejera 2022) that highlights Caulerpaceae, Halimedaceae, Dictyotaceae and Rhodomelaceae as the most prevalent families with the highest species count in reef environments. When considering macroalgae distribution by reef, Cayo Arenas, TE and Serpientes reported more chlorophytes, whereas Bajos Obispo, B. Pera and B. Nuevo were dominated by rhodophytes. This distinction likely arises from the depth variations amongst these reefs, where green algae tend to thrive in the shallower reefs, influencing their development and distribution (Garduño-Solórzano et al. 2005).

In Table 1, these species are newly recorded for Yucatan: *Asteromeniapeltata*, *Botryocladiashanksii*, *Ceratodictyonscoparium* and *Herposiphoniaparca* Setchell. Regarding the newly-reported species in Yucatan, *A.saldanhae* had been previously documented in the Gulf of Mexico in Veracruz by Vázquez-Machorro et al. (2016). *C.scoparium*, on the other hand, had been reported in the Mexican Caribbean around Puerto Morelos and in the Gulf of Mexico by Suárez and Martínez-Daranas (2020). However, for *H.parca*, no records existed, marking it as a new finding for the Mexican Atlantic.

There is a few species shared between reefs indicating a low similarity despite their belonging to the same archipelago. Typically, the similarity between localities increases with proximity, as observed by Nekola and White (1999). Yet, the intricate geographical location and geological history of the Yucatan Peninsula have fostered diverse habitats, resulting in high species richness adapted to specific microenvironments. This variation leads to noticeable differences in the biological composition, even amongst nearby localities (Vilchis et al. 2018).

These reefs serve as significant biodiversity hubs, interconnected with the environmental characteristics of the carbonate shelf where they reside. They receive Gulf of Mexico waters that transport various taxa, contributing to their higher biological diversity, as observed in studies by Horta-Puga et al. 2007, Ortiz-Lozano et al. 2013 and Zarco-Perelló et al. 2013. These factors potentially result in different algal communities compared to other Campeche Bank reefs (Zarco-Perelló et al. 2013).

## Figures and Tables

**Table 1.** Infrageneric Taxa by Localities. Abbreviations: Car = cayo Arenas, BON = Banco Obispo norte, BOS = Banco Obispo Sur, S = Serpientes, TE = Triángulos Este, TN = Triángulos Norte, BN: Bajo Nuevo, BP: Banco Pera. * New distribution report for Mexican Atlantic, ** New distribution reports for Yucatan.

	Infrageneric Taxa:	Car	BON	BOS	S	TE	TN	BN	BP
Num Sp	CHLOROPHYTA								
1	*Anadyomenesaldanhae* Joly & Oliveira		1			1	1		
2	*Anadyomenestellata* (Wulfen) C. Agardh		1	1					
3	*Bryopsispennata* J.V. Lamouroux	1					1		
4	Bryopsispennatavar.secunda (Harvey) Collins & Hervey						1		
5	*Bryopsisplumosa* (Hudson) C. Agardh						1		
6	*Caulerpaambigua* Okamura	1		1			1		
7	*Caulerpachemnitzia* (Esper) J.V.Lamouroux	1							
8	*Caulerpamicrophysa* (Weber van Bosse) Feldmann	1			1	1		1	
9	*Caulerparacemosa* (Forsskål) J. Agardh	1				1			
10	Caulerparacemosavar.macrophysa (Kützing) W. R. Taylor				1				
11	Caulerpasertularioidesf.brevipes (J.Agardh) Svedelius					1			
12	*Caulerpaverticillata* J. Agardh	1	1		1	1			1
13	Caulerpaverticillataf.charoides Weber van Bosse	1				1			
14	*Caulerpawebbiana* Montagne	1			1				
15	*Cladophoraliniformis* Kützing					1			
16	*Cladophoropsismacromeres* W. R. Taylor	1							
17	*Cladophoropsismembranacea* (Bang ex C.Agardh) Børgesen							1	
18	*Dictyosphaeriacavernosa* (Forsskal) Børgesen								1
19	*Halimedadiscoidea* Decaisne				1				1
20	*Halimedaincrassata* (J. Ellis) J. V. Lamouroux			1	1				
21	*Halimedaopuntia* (Linnaeus) J.V. Lamouroux					1	1		
22	*Halimedatuna* (J.Ellis & Solander) J.V.Lamouroux	1							
23	*Penicilluscapitatus* Lamarck				1				
24	*Penicillusdumetosus* (J.V. Lamouroux) Blainville				1				
25	*Phyllodictyonanastomosans* (Harvey) Kraft & M. J. Wynne							1	
26	Rhipocephalusphoenixf.longifolius A.Gepp & E.Gepp				1				
27	*Siphonocladustropicus* (P. Crouan & H. Crouan) J.Agardh			1					
28	*Udoteacaribaea* D. S. Littler & Littler				1				
29	*Udoteacyathiformis* Decaisne	1							
30	*Udoteadixonii* D. S. Littler & Littler			1					
31	*Valoniamacrophysa* Kützing							1	
32	*Valoniaventricosa* J.Agardh		1			1	1		
	PHAEOPHYCEAE								
1	*Canistrocarpuscrispatus* (J.V.Lamouroux) De Paula & De Clerck				1				
2	*Dictyopterisdelicatula* J.V. Lamouroux	1		1					
3	*Dictyotabartayresiana* J.V. Lamouroux			1			1	1	
4	*Dictyotacaribaea* Hörnig & Schnetter			1					
5	*Dictyota ciliolata Sonder ex* Kützing	1					1		
6	*Dictyotahumifusa* Hörnig, Schnetter & Coppejans						1		
7	*Dictyotajamaicensis* W. R. Taylor			1			1		
8	*Dictyota pinnatifida Kützing*			1					
9	*Dictyotapulchella* Hörnig & Schnetter			1	1	1	1		
10	*Feldmanniaindica* (Sonder) Womersley & A. Bailey	1							
11	*Lobophoracanariensis* (Sauvageau) C.W. Vieira, De Clerck & Payri	1							
12	*Lobophoradeclerckii* N.E. Schultz, C.W. Schneider & L. Le Gall	1							
13	*Lobophoravariegata* (J.V. Lamouroux) Womersley ex Oliveira				1				
14	*Padinahaitiensis* Thivy	1							
15	*Padinaperindusiata* Thivy				1				
16	*Padinasanctae-crucis* Børgesen	1							
17	*Sargassumbuxifolium* (Chauvin) M.J.Wynne	1							
18	*Sargassumfurcatum* Kützing	1							
19	*Stypopodiumzonale* (J.V. Lamouroux) Papenfuss		1	1		1	1		
	RHODOPHYTA								
1	*Acanthophoraspicifera* (Vahl) Børgesen								1
2	*Amphiroabeauvoisii* J.V. Lamouroux						1		
3	*Amphiroarigida* J.V. Lamouroux						1		
4	*Amphiroafragilissima* (Linnaeus) J.V. Lamouroux						1		
5	*Amphiroatribulus* (J. Ellis & Solander) J.V. Lamouroux		1	1					1
6	*Antithamnionellabreviramosa* (E.Y.Dawson) Wollaston	1							
7	*Asparagopsistaxiformis* (Delile) Trevisan	1			1				
8	*Asteromeniapeltata*(W. R. Taylor) Huisman & A. J. K. Millar **			1					1
9	*Botryocladiashanksii* E. Y. Dawson **			1					1
10	*Botryocladiaspinulifera* W.R. Taylor & I.A. Abbott								1
11	*Caloglossaleprieurii* (Montagne) G.Martens						1		
12	*Ceramiumnitens* (C. Agardh) J. Agardh			1			1		
13	*Ceramiumvirgatum* Roth							1	
14	*Ceratodictyonintricatum* (C. Agardh) R.E. Norris					1	1		
15	*Ceratodictyonscoparium* (Montagne & Millardet) R.E. Norris **		1	1			1		1
16	*Ceratodictyon variabile (J.Agardh) R.E.Norris*					1			
17	Dichotomariaobtusatavar.major M. J. Wynne							1	1
18	*Dictyurusoccidentalis* J.Agardh				1				
19	*Erythrotrichiacarnea* (Dillwyn) J. Agardh	1							
20	*Flahaultiategetiformans* W.R. Taylor							1	
21	*Galaxaurarugosa* (J. Ellis & Solander) J.V. Lamouroux		1						
22	*Gayliellaflaccida* (Harvey ex Kützing) T. O. Cho & L. J. McIvor	1							
23	*Gelidiellaacerosa* (Forsskål) J. Feldmann & Hamel			1					1
24	*Gelidiumamericanum* (W.R. Taylor) Santelices						1		
25	*Griffithsiaglobulifera* Harvey ex Kützing						1		
26	*Herposiphoniaparca* Setchell*						1		
27	*Hydrolithonfarinosum* (J. V. Lamouroux) Penrose & Y. M. Chamberlain						1		
28	*Hypneaspinella* (C. Agardh) Kützing				1	1	1		
29	*Hypoglossumhypoglossoides* (Stackhouse) Collins & Hervey					1			
30	*Jania adhaerens J*.V.Lamouroux	1							
31	*Janiacapillacea* Harvey			1					
32	*Janiapumila* J.V.Lamouroux	1							
33	*Liagoraceranoides* J.V.Lamouroux	1							
34	*Nitophyllumpunctatum* (Stackhouse) Greville			1					
35	*Parviphycussetaceus* ((Feldmann) J.Afonso-Carrillo, M.Sanson, C.Sangil & T.Diaz-Villa		1		1				
36	*Polysiphoniahavanensis* Montagne							1	
37	*Pterocladiellacapillacea* (S. G. Gmelin) Santelices & Hommersand			1					
38	*Stylonemaalsidii* (Zanardini) K.M. Drew							1	
39	*Wrangeliaargus* (Montagne) Montagne	1							

**Table 2.** Coordinates by locality, sampling point and depth. About the sampling points, letters indicate locality initials and numbers indicate stations within each locality. Banco Obispo (BO) (S or N South or North), Triángulos Oeste (TW) Triangulos Este (TE), Arrecife Serpientes (Ser), Ext (Extra point) Arrecife Banco Pera (BP), BN: Banco Nuevo.

Locality	Sampling point	Decimal Latitude	Decimal Longitude	Depth In Metres
Arrecife Banco Obispo Norte	16_BON	20.50072	-92.20306	11
Arrecife Banco Obispo Norte	1_BON	20.50205	-92.20464	21
Arrecife Banco Obispo Norte	8_BON	20.50371	-92.20200	21
Arrecife Banco Obispo Norte	9_BON_Nocturn	20.49049	-92.20332	20
Arrecife Banco Obispo Norte	10_BON	20.49943	-92.20429	9
Arrecife Banco Obispo Sur	4 _BOS	20.42059	-92.22333	13
Arrecife Banco Obispo Sur	2_BOS	20.42529	-92.22865	25
Arrecife Banco Obispo Sur	1_BOS	20.42343	-92.22603	18
Arrecife Banco Obispo Sur	3_BOS	20.42133	-92.22256	13
Arrecife Banco Pera	BP	20.72669	-91.93481	24
Arrecife Cayo Arenas	Ext 001	22.11670	-91.39809	1.5
Arrecife Cayo Arenas	Point 02	22.12064	-91.40636	9.3
Arrecife Cayo Arenas	point 01	22.12092	-91.38831	9
Arrecife Cayo Arenas	point 03	22.11557	-91.40161	6.8
Arrecife Cayo Arenas	Point 06	22.11668	-91.39764	7.5
Arrecife Cayo Arenas	Ext002	22.11558	-91.40008	1
Arrecife Cayo Arenas	Point 10	22.11181	-91.39808	13
Arrecife Cayo Arenas	Point 32	22.11086	-91.37644	11.9
Arrecife Cayo Arenas	Point 09	22.11352	-91.38397	12
Arrecife Cayo Arenas	Point 34	22.11353	-91.37839	18.5
Arrecife Cayo Arenas	Point 31	22.11236	-91.37344	15.5
Arrecife Cayo Arenas	Point 30	22.12114	-91.39925	20.6
Arrecife Cayo Arenas	Point 28	22.11894	-91.37289	6.6
Arrecife Cayo Arenas	Ext 07	22.11868	-91.40374	8
Arrecife Serpientes	SER-01	21.43936	-90.47292	9
Arrecife Serpientes	SER-02	21.43478	-90.45086	9.9
Arrecife Triángulo Este	15_TE	20.89435	-92.23953	15
Arrecife Triángulo Este	3_TE	20.90456	-92.23322	11
Arrecife Triángulo Este	15_TE	20.90833	-92.21313	28
Arrecife Triángulo Oeste	12_TW	20.95528	-92.31086	18
Arrecife Triángulo Oeste	7_TW	20.95816	-92.30516	18
Arrecife Triángulo Oeste	8_TW	20.95856	-92.30441	15
Arrecife Triángulo Oeste	1_TW	20.95887	-92.30717	0
Arrecife Triángulo Oeste	11_TW	20.96036	-92.30776	11
Arrecife Triángulo Oeste	2_TW	20.96221	-92.31128	15
Banco Nuevo	BN	20.54561	-91.87944	24
